# Control of MRSA infection and colonisation in an intensive care unit by GeneOhm MRSA assay and culture methods

**DOI:** 10.1186/1471-2334-9-137

**Published:** 2009-08-24

**Authors:** Claudia Dalla Valle, Maria Rosalia Pasca, Debora De Vitis, Federico Capra Marzani, Vincenzo Emmi, Piero Marone

**Affiliations:** 1 Laboratorio di Batteriologia e Micologia, Area Infettivologica, Fondazione IRCCS S. Matteo, Pavia, Italia; 2 Dipartimento di Genetica e Microbiologia, Università degli Studi di Pavia, Italia; 3 Rianimazione I, Fondazione IRCCS S. Matteo, Pavia, Italia

## Abstract

**Background:**

Methicillin-resistant *Staphylococcus aureus *(MRSA) is one of the major nosocomial pathogens. Due to the diffusion of MRSA strains in both hospital and community settings, prevention and control strategies are receiving increased attention. Approximately 25% to 30% of the population is colonised with *S. aureus *and 0.2% to 7% with MRSA. The BD GeneOhm MRSA real-time PCR assay offers quicker identification of MRSA-colonised patients than do culture methods.

**Methods:**

Ninety-five patients admitted to the Intensive Care Unit of IRCCS Policlinico San Matteo of Pavia (Italy) for a period > 24 h were screened for MRSA colonisation with both the culture method and the GeneOhm assay.

**Results:**

Of the 246 nasal swabs collected from 95 patients, 36 samples were found to be positive by both methods (true-positive). 30% of colonised patients had developed the MRSA infection.

**Conclusion:**

Our results show that the GeneOhm MRSA assay is a valuable diagnostic tool for detecting MRSA quickly in nasal swabs. This study confirms that colonisation represents a high risk factor for MRSA infection, and that good MRSA surveillance in an Intensive Care Unit is therefore an excellent way to prevent MRSA infection.

## Background

Methicillin-resistant *Staphylococcus aureus *(MRSA) is a nosocomial pathogen that is conspicuously responsible for increased morbidity and mortality, as well as for prolonged hospitalization. As such, it substantially increases hospital and health system costs [1,2].

MRSA originates from the introduction of a large mobile genetic element called staphylococcal cassette chromosome *mec *(SCC *mec*) into a methicillin-susceptible *S. aureus *strain. Methicillin-resistance is conferred by the *mecA *gene, which encodes a penicillin-binding protein (PBP2A) with decreased affinity for β-lactam antibiotics, and which forms part of the mobile genetic element [3].

MRSA infections have recently become the focus of intense media attention. In 2005, the United States press described MRSA as the "super bug", on the basis that it killed more people than did AIDS [4]. Due to the diffusion of MRSA in both hospital and community settings, prevention and control strategies are receiving increased attention. Multiple infection control practices, including hand hygiene, identification of MRSA carriers, patient-decolonization, and environmental decontamination, are used in combination to prevent nosocomial infections. Contact isolation of MRSA carriers also prevents transmission within the hospital [5].

Approximately 25% to 30% of the population is colonised with *S. aureus *and 0.2% to 7% with MRSA; consequently, not only infected patients, but also colonised patients, represent the most important reservoir of MRSA in health-care facilities. Nasal MRSA colonisation can serve as a source for transmission and is also considered a risk factor for subsequent infection [6]. Consequently, periodic nasal swab collection contributes positively to the containment of MRSA in high risk areas.

The combined use of surveillance cultures and barrier precautions produces cost savings for hospitals. The cost of caring for MRSA-infected patients greatly exceed that of screening programmes.

Because it is more sensitive than culture methods, PCR identifies MRSA-colonised patients more quickly [7]. Culture-based detection of MRSA with traditional media requires 48 to 96 h for results. In contrast, new real-time PCR-based methods allow differentiation of methicillin-sensitive *Staphylococcus aureus *(MSSA), MRSA, and coagulase-negative Staphylococci within a few hours [8-11]. Such tests promote an early appropriate antibiotic selection, and they reduce mortality, the length of hospitalization, and the costs associated with infections caused by these bacteria [12]. Furthermore, the BD GeneOhm MRSA real-time PCR assay, formerly called the IDI-MRSA assay (BD Diagnostics, San Diego, CA), identifies MRSA-colonised patients in as little as 2 h [9]. This multiplex real-time PCR amplifies an *S. aureus*-specific target sequence near the staphylococcal cassette chromosome *mec *(SCC *mec*) insertion site and the *orfX *junction in MRSA, and thereby differentiates between MSSA and MRSA [9,11].

We evaluated the diagnostic performance of the GeneOhm MRSA assay for the detection of MRSA from nasal swab from patients hospitalized in an Intensive Care Unit.

## Methods

### Patients

The study period was from 10^th ^July to 23^rd ^October 2007. The "Servizio di Anestesia e Rianimazione I" of the IRCCS Policlinico San Matteo, Pavia, is one of the hospital's two general Intensive Care Units. The unit in question comprises 10 sleeping accommodations and usually admits about 450 patients yearly. More than half of the population are surgical patients; about 30% are admitted from medical wards within the hospital or from the emergency room for non-surgical reasons or on account of trauma.

The unit isolates patients upon MRSA identification because even more MRSA colonisation without infection can serve as an MRSA source and hence endanger other patients. MRSA-colonised patients without infection are not subjected to antibiotic or colonisation therapy.

Ninety-five patients admitted to the Intensive Care Unit of IRCCS Policlinico San Matteo, Pavia, for a period > 24 h were screened for MRSA colonisation. Nasal swab collection was performed upon admission and twice-weekly thereafter. The swab was introduced into the anterior nare and rotated five times. Then the nasal swab was inserted into a tube containing 1 ml Tryptic Soy Broth (TSB, Oxoid) with NaCl 6.5%. The specimens were transported at room temperature and processed with culture and molecular methods.

### Culture methods

All isolates were processed by standard laboratory methods. The nasal swab was used to directly streack (about 100 μl) the samples onto Mannitol-salt-agar (Biomerieux) and Columbia blood agar (Biomerieux). The plates were incubated at 35°C for 24–48 h. A putative MRSA colony was confirmed by Gram stain, catalase test, and tube coagulase. Methicillin-resistance was tested by means both of Mueller-Hinton agar containing 6 mg/l oxacillin and 4% NaCl, and of the cefoxitin disk diffusion method, in accordance with the CLSI protocol [13].

### Real-time procedure

The swabs were also processed with the real-time PCR-based BD GeneOhm MRSA Assay (Becton Dickinson Diagnostics), in accordance with the manufacturer's instructions. Our use of exclusively nasal swabs was determined by the manufacturer's recommendation and by the fact that the nostrils are indeed the main site of *S. aureus *colonization.

The procedure was very easy: the nasal swab was placed in a tube with the buffer, and the resulting suspension was transferred to a lysis tube for DNA extraction. Subsequent addition of kit's molecular reagents was followed by real-time PCR. The entire process run-time was about 2 hours.

A positive control (supplied with the kit) and a negative control (reaction without template) were included in each run.

### Data analysis

GeneOhm MRSA results were compared with those obtained from the culture method. A sample found to be positive under both GeneOhm assay and the culture method was defined a true-positive result. A true-negative result was one that proved negative under both methods. A sample that was positive under GeneOhm assay and negative under culture method was defined as false-positive.

The per sample cost of the molecular method was approximately € 40. Calculated from multiple applications, this figure is an average, and takes into account the kit reagents, the consumable materials, and the kit controls (positive and negative), which were invariably processed with each sample. The procedure time for this method was about 2 hours.

The culture method cost about € 4, a figure that comprised the use of selective plates, biochemical tests, and consumable materials. Procedure time was 36–48 hours.

The study procedure was approved by IRCCS Policlinico San Matteo, Pavia, Italy.

## Results and discussion

Ninety-five patients admitted to the Intensive Care Unit (I) of IRCCS Policlinico San Matteo of Pavia from 10^th ^of July to 23^rd ^of October 2007 for a period > 24 h were screened for MRSA colonisation. Altogether, 246 nasal swabs from 95 patients were processed by both the molecular and the culture methods. Forty seven patients were hospitalised for a period < 3 days and underwent nasal swab only once; 17 patients were nasally swabbed twice, 9 patients three times, and 22 patients 4 or more times.

### MRSA detection by culture method

A total of 246 nasal swabs were processed with the culture method, as described above. Only 36 samples were positive for MRSA colonisation (14.6%).

### MRSA finding by GeneOhm assay and comparison with culture method

During our study, 246 nasal swabs from 95 ICU patients were tested using PCR and conventional methods. Table 1 shows fifty-six nasal swabs as positive for MRSA colonisation under GeneOhm assay (22.76% of the total 246 nasal swabs performed).

**Table 1:** Comparison between results obtained by the culture and molecular methods

Gene Ohm assay	Culture method		
	
	Positive	Negative	Total
Positive	36	20	56

Negative	0	190	190

Total	36	210	246

Of these, 36 samples (14.6%) were positive under culture method (true-positive), while 20 (8.8%) were positive under GeneOhm assay and negative under culture method (false-positive).

Within the false-positive group, 5 patients yielded only a single nasal swab either because they died in, or were transferred from the hospital. A further 12 false-positive results derived from 3 patients that had previously been found to be positive to MRSA colonisation under culture method. Arguably, the false-positive MRSA colonisation status of these patients could be due to the molecular method's greater sensitivity to what could have been very low bacterial concentrations. In other words, it is possible that some or all of the given 12 false-positive were in fact true-positive results.

In the present study, GeneOhm™ MRSA assay gave no false-negative, and only 20 false-positive results. A total 190 (77.23%) samples were found to be negative under both methods. Thirteen cases (5.2%) were not resolved by GeneOhm assay, but proved to be positive under repeated assay.

Agreement between the molecular and culture methods was 91.8% (226/246). Comparatively, GeneOhm assay was significantly more sensitive than the culture method: the former demonstrated sensitivity of 100% (36/36) and specificity of 90,4% (190/210). Our choice of collecting exclusively nasal swabs was based on the fact that the anterior nare is the primary basin for *S. aureus*. Previous studies [7,14] have shown that nose-only specimens are 90% sensitivity for MRSA-detection purposes, while combined nose and groin samples are 88% sensitive, and skin or other superficial sites 76.5%. Exclusively nasal sampling would appear to be adequate. Moreover, our data indicated that nasal surveillance captured 100% of MRSA colonisation, a finding which enhances the case for this method as a sound basis for MRSA detection.

As shown in previous studies and in this work, BD GeneOhm™ MRSA assay is both specific and sensitive [1,10,14-17]. Similar results regarding the sensitivity of the GeneOhm™ MRSA assay for single throat swabs have already been described by Rossney *et al*. (2007) [16] (89.0% sensitivity and 99.0% specificity) and van Hal *et al*. (2007) [17] (90.0% sensitivity and 96.0% specificity). In a recent study by Svent-Kucina *et al*. (2009) [11], the GeneOhm™ MRSA assay protocol was modified during the specimen preparation step by the addition of an extra washing step, followed by the pooling of up to 3 samples per patient (nose, skin, with or without throat) at the lysis step. The sensitivity and specificity rates of the modified assay in comparison with those of conventional culture were respectively 94.3% and 99.2%.

In a recent study, Boyce *et al*. (2008) [18] compared the GeneOhm MRSA assay with the CHROMagar MRSA assay (BD Diagnostics) for the detection of MRSA in 286 nasal surveillance specimens; the GeneOhm assay proved to be both faster and more sensitive.

All these examples agree with our results on the sensitivity and specificity of the molecular method utilised.

### Infection rate versus colonisation rate

Ten patients were true-positive under both methods; 2 of them were MRSA carriers on admission, while 8 were colonised in ICU: 3 patients after 4 days, 4 after 5–7 days and 1 after 30 days. The colonisation rate was 10.5% (10/95), but it increases to 16.8% (16/95) if we add 6 false-positive patients that were MRSA carriers on admission. All 10 patients were isolated upon MRSA identification.

Only 3 (30%) MRSA colonised patients developed MRSA infection. Specifically, after 7 days of colonisation, 2 patients respectively showed an MRSA infection from a surgical wound, and pneumonia; after 10 days of colonisation, the 3^rd ^patient developed central venous catheter-related sepsis.

### Disadvantages and benefits of the molecular method

The molecular assay here described is performed directly from the sample (e.g. nasal swab). Previous real-time PCR assays have demonstrated a capacity to detect MRSA rapidly in culture [9]. Reischl *et al*. (2000) [19] reported a duplex assay for *mecA *and the *S. aureus*-specific *sa442 *genome. They reported 100% sensitivity and specificity for the detection of MRSA in "pure colonies". The critical advantage offered by the GeneOhm MRSA assay is that it circumvents pure colony isolation and thus enables direct use of specimens.

However, none of the assays described before [9,19] was able to differentiate methicillin-resistant *S. aureus *from methicillin-resistant coagulase negative *Staphylococcus *spp. in primary specimens that simultaneously host both of these organisms, such as occurs in anterior nares specimens. In the GeneOhm assay, the use of primer sequences for the *SCCmec *and *orfX *regions generates an MRSA-specific amplicon, which in turn is detected by a complementary molecular beacon probe.

Reliable and rapid detection of MRSA-colonised patients is essential for the successful prevention and control of MRSA outbreaks in hospital, and hence for overall patient care and hospital infection control. The BD GeneOhm™ MRSA assay's speed (amplification time of 2 h versus 36–48 h for the culture method) gives it a critical advantage. Timely identification of MRSA colonisation allows the isolation and treatment of affected patients and thus is essential for the prevention of MRSA outbreaks. Cunningham *et al*. (2007) [20] demonstrated a reduction in MRSA transmission incidence from 13.9/1,000 patient days under phenotypic (culture-based) MRSA surveillance to 4.9/1,000 patient days under PCR screening.

MRSA prevalence and incidence within an institution are the major determinants of the number of patients that need to be screened in order to prevent one infection. Molecular methods cost approximately 10 times more per test than conventional methods (€ 40 *versus *€ 4), and infection control programs remain poorly resourced. However, rapid screening is able to save on patient preventive isolation costs (if instituted) and on costs associated with the prevention of hospital-acquired MRSA infections [21].

Further research into the cost effectiveness of molecular testing is needed.

## Conclusion

The growing threat of MRSA is increasingly apparent both to the public and to health care providers. MRSA infections are yet another major factor in the current spiralling of health care costs. It is imperative that clinicians have a high index of suspicion for MRSA so that the initiation of the appropriate antibiotic therapy is prompt [1].

Our results with the GeneOhm MRSA assay show that it is an accurate and rapid way to detect MRSA colonisation. This assay can be performed by a microbiologist technician with minimal additional training and allows same-day results.

This study also confirms that colonisation is a high risk factor for MRSA infection, and consequently that good MRSA surveillance in an Intensive Care Unit is an excellent way to prevent MRSA infection.

## Competing interests

The authors declare that they have no competing interests.

## Authors' contributions

CDV, MRP: data analysis, manuscript preparation; DDV: real-time procedures; FCM, VE: collection of samples and clinical data, data analysis; PM: design of the study, data analysis, manuscript preparation.

## Pre-publication history

The pre-publication history for this paper can be accessed here:

http://www.biomedcentral.com/1471-2334/9/137/prepub
